# Exploring the Specifications of Spatial Adjacencies and Weights in Bayesian Spatial Modeling with Intrinsic Conditional Autoregressive Priors in a Small-area Study of Fall Injuries

**DOI:** 10.3934/publichealth.2016.1.65

**Published:** 2016-03-04

**Authors:** Jane Law

**Affiliations:** School of Public Health and Health Systems, University of Waterloo, ON, Canada

**Keywords:** spatial structure, variable weights, equal weights, adjacent matrix, Bayesian, spatial epidemiology, WinBUGS, neighbourhood structure

## Abstract

Intrinsic conditional autoregressive modeling in a Bayeisan hierarchical framework has been increasingly applied in small-area ecological studies. This study explores the specifications of spatial structure in this Bayesian framework in two aspects: adjacency, i.e., the set of neighbor(s) for each area; and (spatial) weight for each pair of neighbors. Our analysis was based on a small-area study of falling injuries among people age 65 and older in Ontario, Canada, that was aimed to estimate risks and identify risk factors of such falls. In the case study, we observed incorrect adjacencies information caused by deficiencies in the digital map itself. Further, when equal weights was replaced by weights based on a variable of expected count, the range of estimated risks increased, the number of areas with probability of estimated risk greater than one at different probability thresholds increased, and model fit improved. More importantly, significance of a risk factor diminished. Further research to thoroughly investigate different methods of variable weights; quantify the influence of specifications of spatial weights; and develop strategies for better defining spatial structure of a map in small-area analysis in Bayesian hierarchical spatial modeling is recommended.

## 1. Introduction

In small-area ecological studies using a Bayesian approach, spatial dependence is accounted for at the prior level, often using the intrinsic conditional (Gaussian) autoregressive (ICAR) model as a means to capture the effects of unobserved spatially-structured latent covariates or measurement errors. Data for each area are then assumed independent conditional on the spatial ICAR (or proper CAR) model. Within the framework for estimating risks and identifying risk factor(s), the ICAR model is used as part of a convolution prior for the (log) relative risk [1],[2]. The convolution prior consists of a spatially-structured random effects term (S) and an unstructured random effects term (U) modeling the underlying (log) relative risks. It enables each area to borrow strength globally through the prior specification of a normal distribution for the unstructured random effects, and locally from neighboring observations through the prior specification of the ICAR model for spatial random effects. As a result, estimated risks are smoothed towards a combination of global (through U) and local (through S) risks. It is the data that determine the relative contribution of each of them. Smoothing of estimated risks with the ICAR model is helpful for stabilizing risks [3], but over-smoothing can be a related issue [4]. Unexpected changes in estimates of risks when compared to standardized ratios may reflect inappropriate smoothing.

Prior specifications of the ICAR model in essence contain two pieces of spatial information about spatial structure: adjacencies and weights. Adjacencies information specifies for each area, the set and number of areas adjacent to it. Weights information specifies the weight assigned to each pair of adjacent or neighboring areas. In terms of an adjacency matrix (W), each of the elements (W_ij_) in the matrix, depicts the spatial closeness of areas i and j and quantifies the dependence between the outcomes of the two areas. Conventionally, in small-area analysis, two areas are specified as adjacent or neighbors by binary weights (i.e., one for neighbors and zero for non-neighbors), and W is standardized such that its rows sum to 1, so effectively applying a weighted average of neighboring values into the analysis.

The first piece of spatial information about adjacencies is important in terms of its accuracy in reflecting true adjacencies but has rarely been discussed. In small-area analysis, adjacency is most commonly defined by queen-based contiguity (two areas sharing a common boundary point or vertex are considered as neighbors). Other definitions include rook, bishop, and distance. Often, whether two areas are captured correctly as adjacent, i.e., sharing a common vertex or line, is assumed to be determined by the software (e.g., GeoDa, WinBUGS, R) used to generate the adjacencies information from a digital map. Nevertheless, our concern in this study is not about the capability of the software, but the quality of the digital map itself, in enabling true adjacencies information to be generated. The latter has often been neglected. Analysts would not usually check the digital map for correctness of the adjacencies information generated before using it for analyses.

The second piece of spatial information about weights can be more of an issue than adjacencies. Prior specifications of weights play an important role in the smoothing process because the risk estimated for a given small area is conditionally dependent on the risk estimates from its adjacent areas and the weight (to be borrowed) assigned to each of its adjacent areas. A common choice is to set all the weights fixed and equal to one (refers to as equal weights below), probably for computational simplicity. The (Gaussian) ICAR distribution that models the (log) relative risk of an area has a mean s_i_ that is linked to the mean of the neighboring (j) risks via an autoregressive formulation of ∑_j_w_ij_s_j._ The method of equal weights assumes isotropy of correlation and constant variance of heterogeneity, disregarding the varying strengths of relationship between areal units. Depending on the structure of the data, in terms of level of aggregation and numbers of the population-at-risk between areas, using equal weights disregard can be an issue [5]. Logically, pair of adjacent areas should have correlated random effects only, and strength should be borrowed from each other for analysing risks only, if they are somehow similar. Borrowing information equally from all neighboring areas disregarding their similarity or dissimilarity, in terms of population-at-risk heterogeneity is questionable. We question for instance whether borrowing of information from a neighboring area that has extremely low population-at-risk (seniors in our case study) is appropriate.

Apart from equal weights, methods of variable weights have been studied in spatial autoregressive modeling. Weighting has been specified deterministically in terms of the distance between area centroids for analysing disease rates but was not found better than equal weighs; however, variable weights based on population density to enhance rate stabilization in rural areas was recommended [6]. Deterministic approaches also include using a small number of regression parameters to determine the set of weights for capturing localized spatial correlation and identifying risk boundaries in research of boundary detection [7]. Variable weighting has also been studied stochastically through modeling each weight as a separate unknown parameter. For example, weights have been modeled using a Bernoulli distribution as a set of binary random quantities in research of areal wombling, and estimated by logistic regression using a covariate that measures similarity or dissimilarity between areas with hierarchical modeling [8]. Further studies replaced logistic regression with other types of model such as a second stage CAR prior [9]. These stochastic models that introduce a large number of unknown (partial autocorrelation) parameters of weights through adding an additional parameter of probability, p_ij_, that the weight, w_ij_, equals 1 for each pair of neighbors i and j (w_ij_|p_ij_ ∼Bernoulli (p_ij_)) showed critical problems of overparameterization and parameter identifiability [10]. They were found computationally expensive to update and require the development of software to run as there is no existing commercial software that can fit these models. In practice, they have been rarely applied and are not suitable for analysing datasets that involve a large number of areal units.

In this study, we explore a relatively simple deterministic approach for applying variable weights. The approach does not introduce additional calculations (e.g., distances) or parameters of regression or weights to the conventional ICAR equal-weights model and can be implemented using existing freeware such as WinBUGS. The study was motivated during a joint research on seniors' falls with the Region of Wellington-Dufferin-Guelph (WDG) Public Health unit in Ontario. The joint research aims to estimate risks, locate areas that have relatively high risk, and identify risk factors of falling injuries among seniors (people age 65 and over) in the WDG region [11]. Falls is the leading cause of injury-related hospitalization in Canada. The average annual rate of fall in the WDG region is 1,838 per 100,000, which is higher than the 2007 average rate in Ontario [12]. The research applied ICAR with convolution prior, and was conducted at the census dissemination area (DA) level. DA is the smallest census area that covers the entire country of Canada. As with almost, if not all, census areas, DAs in the WDG region are not regularly spaced nor of similar size.

We report below our methods of research and results, followed by discussion and conclusion with recommendations for further research.

## 2. Method

### 2.1. Data and study region

**Figure 1.**
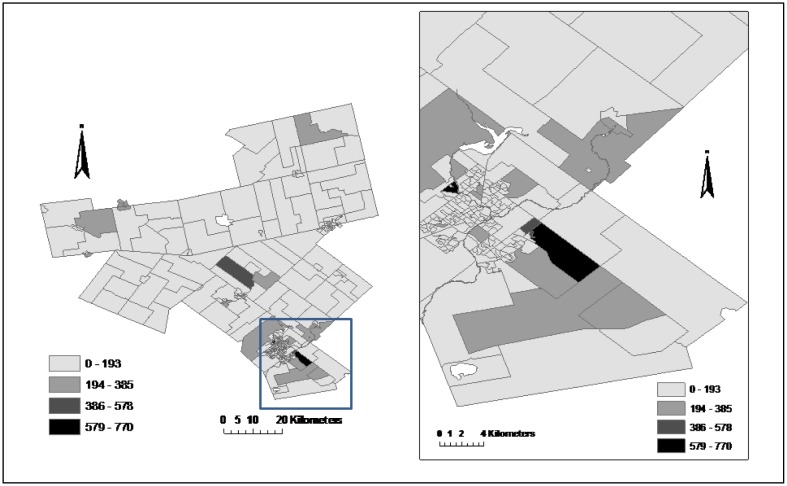
Population per square kilometre of seniors age 65 and over in 2006, Region of Wellington-Dufferin-Guelph, at the census dissemination area level.

The Region of WDG Public Health Unit provided the census data and digital map of DAs of the study region in shapefile format. The study region, which has an area of 4,242 sq. kilometres and a population of 238,326 (according to the 2006 Census), includes the counties of Wellington, Dufferin, and city of Guelph, about 100 kilometres west of Toronto. It has experienced aging of population with some parts having apparently more seniors of age 65 and over (figure 1). Aside from non-communicable diseases, injury has been the leading cause of death in the region. About 80% of injury-related hospitalizations were the results of falls in seniors. The rate of fall-related injuries was nine times greater than among seniors than the rest of the population. 57% of seniors' falls occurred at home.

The original data source of hospitalization due to falls (ICD codes W00-19) is the Ontario Provincial Health Planning database (obtained through the Region of WDG health unit). Data contain counts of falls separated by female and male and in five age groups (65–69, 70–74, 75–79, 80–84, 85+), i.e., 10 strata, between 2002 and 2006 in each of the 308 DAs in the study region. The percentage of DAs that have zero count of seniors' falls is 0.09. The total number of seniors' falls in the region within the study period is 2,712.

Covariates for regression analysis to identify potential risk factors of falls are restricted by those available from the 2006 Census. Totally, 11 covariates related to demographics, services, and housing were tested: 1) population density; 2) median income of population 15 years and over before tax; 3) seniors with low education; 4) seniors living alone; 5) person spent any number of hours providing unpaid care to seniors; 6) rented occupied private dwellings; 7) occupied private dwellings constructed before 1946; 8) occupied private dwellings constructed before 1960; 9) occupied private dwellings constructed before 1980; 10) occupied private dwellings requiring repairs; and 11) apartment building. They are in percentage unless stated otherwise above.

### 2.2 Bayesian hierarchical ICAR modeling

We adopt the hierarchical spatial model proposed by Besag et al. [1] that has been commonly used for epidemiological studies with small-area data. Assuming that injury from falls is rare, the number of cases of injury from falls in area i and age-sex strata k, Y_ik_, where i = 1, …, n, and k = 1, …, K, can be represented by a Poisson model:

$${\text{Y}}_{\text{ik}}\sim \text{Poisson}\left({\text{N}}_{\text{ik}}{\text{p}}_{\text{ik}}\right),$$

where N_ik_ and p_ik_ denote the population and (unknown) probability of injury from falls, in area i and age-sex strata k, respectively.

This Poisson model involves a large number (i x k) of unknown probabilities or parameters of p_ik_. Because our interest is to analyse area-level risks for the entire group (rather than individual age or sex groups) of seniors and the effects of risk factors (covariates) across all the strata, the model can be simplified by making proportionality assumption that p_ik_ = r_i_ x z_k_, where r_i_ and z_k_ represent the relative risk in area i (to be estimated within the model) and z_k_ the reference rate in strata k (to be estimated from the data), respectively. The model for counts of injury falls becomes:

$${\text{Y}}_{\text{i}}\sim \text{Poisson}(\lambda_{\text{i}}),$$ (1)

where λ_i_ = E_i_ x r_i_, and E_i_ denotes the expected count of injury from falls in area $i=\overset{K}{\underset{k=1}{\sum }}N_{ik}z_{k}$. To model r_i_, Equation (1) can be written as:

$$\text{log}\left[\lambda_{\text{i}}\right]\;=\text{ log}\left[{\text{E}}_{\text{i}}\right]\;+\;\alpha \;+\;\beta_{\text{1}}{\text{X}}_{\text{1}}{,}_{\text{i}}+\;\ldots \;+\;\beta_{\text{q}}{\text{X}}_{\text{q}}{,}_{\text{i}}+{\text{ S}}_{\text{i}}+{\text{ U}}_{\text{i}},$$ (2)

where α is an intercept term; β_1_, β_2, ...,_ β_q_ are regression parameters; and X_1_, X_2, ...,_ X_q_ are a set of covariates. S_i_ and U_i_ are spatially-structured random effects and unstructured random effects, respectively, specified by:

$$U_{i}\sim Normal\;(0,\;\upsilon_{u}),\text{ where }\nu_{\text{u}}\text{is the variance of U};\text{ and}$$ (3)

$$S_{i}|s_{-i}\sim Normal\;(\frac{\sum_{j}w_{ij}s_{j}}{w_{i^{+}}},\;\frac{\upsilon_{s}}{w_{i^{+}}})$$ (4)

where ν_s_ is the variance of S, w_ij_ is the weight for area i and area j; and w_i+_ is the sum of weights for area i from all of its neighbors.

Equation (2) assumes a linear relationship between the outcome and explanatory variables. Assuming linearity is the most common approach for regression analysis and considered sufficient for the purpose of this study on spatial structure. The expected counts, E_i_s, were computed by indirect standardization by sex and the five age groups. The number of DAs that have zero expected count is one. The ICAR prior (Equation 4) is a special case of the proper CAR model. It assumes that the spatial dependency parameter, ρ, in the proper CAR model takes its maximum value of one. The relationship of this parameter between the CAR and ICAR models has been discussed in the literature [13],[14]. ICAR was implemented in WinBUGS using its “car.normal” distribution. Arguments input for the distribution contain the adjacencies and weights information for each area. The distribution allows specification of an arbitrary weight vector to define pairwise proximity between areas, including weights by population or expected counts; however, users are required to specify unnormalised weights, wij, for each pair of neighbors. WinBUGS then internally fixes ρ to its maximum value of one through normalising the weights.

We used an improper uniform (dflat in WinBUGS) prior for α, and a normal distribution with a mean of 0 and variance of 10,000 for βs. For the inverse of ν_u_ and ν_s_, i.e., precision of U and S, we specified a Gamma (0.5, 0.0005) prior [15]. This hyperprior distribution is vague, so it allows the model to get the most information from the data. It assumes that the standard deviation of S or U is centred around 0.05 with a 1% prior probability of being smaller than 0.01 or larger than 2.5. For sensitivity analysis, we also tested the Gamma (0.001, 0.001) hyperprior for the precision of U and S.

### 2.3 Adjacency

Queen-based contiguity was used to define adjacencies. It is sensitive to deficiencies of a digital map in providing true adjacencies due to issues with common boundary points, which we aim to investigate. We used GeoDa [10] to generate adjacencies information from the digital map, and the “USGS Adjacency For WinBUGS Tool” [16] to generate another set of adjacencies information for reference. Note that our goal has been to assess the quality of the digital map rather than software packages in generating true adjacencies information. We mapped the shapefile using GIS to obtain manually adjacencies for each of the 308 DAs and prepared a list of correct (true) adjacencies. The list was compared with those generated by the packages.

### 2.4 Equal weights versus variable weights

Using the traditional method of equal weights with the correct adjacencies information, we identified the covariates that were associated with seniors' falls using Equation (2). To control for convergence issues in Bayesian spatial analysis [17], the 11 covariates were first tested individually (i.e., with one explanatory variable in Equation (2) at a time). All covariates with nonzero regression coefficients at the 95% credible interval (C.I.) were considered as significant and included in Equation (2) for multivariate analyses with variable or equal weights as described below.

Based on Equation (2) that contains all the significant covariates identified by equal weights, we compared three variable weights approaches: 1) weights by population count of seniors, i.e., population-at-risk, 2) weights by population density of seniors, and 3) weights by expected count of seniors' falls.

By variable weights, we measured similarity between two neighboring areas based on a variable in determining the weight to be specified, and thus the extent of information to be borrowed from each neighbor. Similarity between each pair of neighboring areas was initially measured using demographic data, specifically, population (at risk) in the two areas according to their product of population counts, i.e., Population_i_ x Population_j_, where i and j are the two adjacent areas. In the presence of spatial correlation, the more seniors (population-at-risk) in an area and its neighboring area(s), the more information there is about this area's spatial random effects.

We further explored the method of variable weights by using the variable of population density of seniors (given by the product of the population density of two adjacent areas), and also the variable of expected count of seniors' falls (given by the product of the expected counts of seniors' falls in two adjacent areas, i.e., weight = E_i_E_j_). The latter (expected count) but not the formers (population-at-risk count or population-at-risk density), adjusts for varying risks of different sex and age groups in each area. This adjustment is useful especially because our data show that seniors who were 85 or above has a higher fall rate compared to other age groups such as 50-65. Such variation of rates has often been found in epidemiology studies where certain age groups tend to have higher rate of the disease or adverse health outcome under study. Specifying the weight of two adjacent areas based on their expected counts reflects the situation of varying age groups of population-at-risk in each area and varying risks of different age groups in the study region.

Comparisons of the above three methods of variable weights were made using the deviance information criterion (DIC) [18]. The result with the smallest DIC (by a difference of greater than five) indicates better model fit. This allows us to identify which of the three variables provided the weighting that outputs the best model for the dataset.

Results of variable weights from the variable (population, population density, or expected count) that gives the best model fit were compared with those from equal weights (i.e., w_ij_=1) based on Equation (2) that contains all the covariates identified. Comparisons were made in terms of model fit (by DIC); model parameters; relative risks; smoothing effects; spatial random effects and their standard errors; and probabilities of relative risk greater than one. They provide insights into the effects of using traditional equal weights versus weights by the (best) variable identified.

## 3. Results

**Figure 2.**
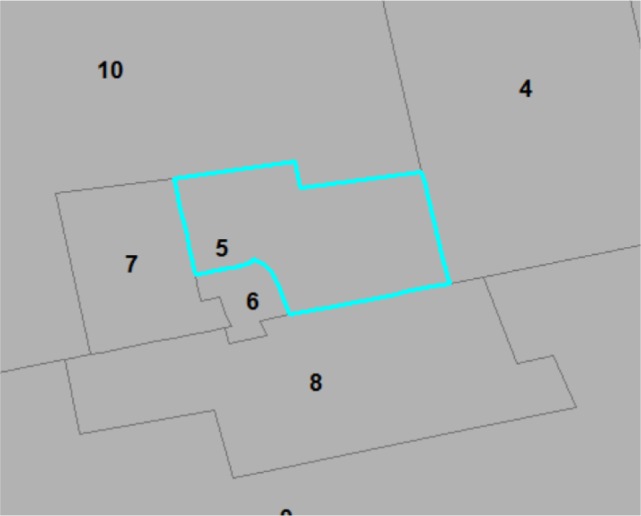
Discrepancies between generated and true adjacencies. Note: Area #5 has five neighbors, but only one of its neighbors (Area #8) has a boundary point that has the same coordinates with a boundary point of Area #5.

Results of adjacencies information from both software packages contain errors when compared to the correct adjacencies information, but the errors are not exactly the same. The total numbers of neighbors found using GeoDa and the USGS program are 1,682 and 1,726, respectively. The (correct) total number of neighbors should be 1,734. As an example, GeoDa reported one neighbour for an area (Area #5 in Figure 2), but it actually has five neighbors, which was reported correctly by the USGS program. Some common boundary vertices of adjacent polygons have different (although close) coordinates recorded in the digital map (i.e., as two different points), which could have led to the incorrect adjacencies information.

Based on Equation (2) with equal weights, we identified significant covariates for multivariate analysis. Of all the 11 covariates tested, “percentage of occupied private dwelling requiring repairs” (X_1_) and “median income of population 15 years and over before tax” (X_2_) have nonzero regression coefficients at the 95% credible level. Categorising whether a dwelling is requiring repairs or not could involve a subjective decision, so it would be better to re-express the data values of “percentage of occupied private dwelling requiring repairs” in percentile and rerun the regression analysis using its quintiled categories (i.e., rather than using it as a continuous variable). Another advantage of using quintiled categories is that the regression analysis then does not make an assumption about linear association. Our final regression model (Equation (5)) therefore contains the “percentage of occupied private dwelling requiring repairs” in percentile (X_1:25_, X_1:50_, and X_1:75_ for the 25^th^, 50^th^, and 75^th^ percentile, respectively) and “median income of population 15 years and over before tax”, X_2_:

$$\text{log}\left[\lambda_{\text{i}}\right]\;=\text{ log}\left[{\text{E}}_{\text{i}}\right]\;+\alpha +\;\beta_{\text{1}}{\text{X}}_{\text{1}:\text{25}}{,}_{\text{i}}+\;\beta_{\text{2}}{\text{X}}_{\text{1}:\text{5}0}{,}_{\text{i}}+\;\beta_{\text{3}}{\text{X}}_{\text{1}:\text{75}}{,}_{\text{i}}+\;\beta_{\text{4}}{\text{X}}_{\text{2}}{,}_{\text{i}}+{\text{ S}}_{\text{i}}+{\text{ U}}_{\text{i}},$$ (5)

**Figure 3.**
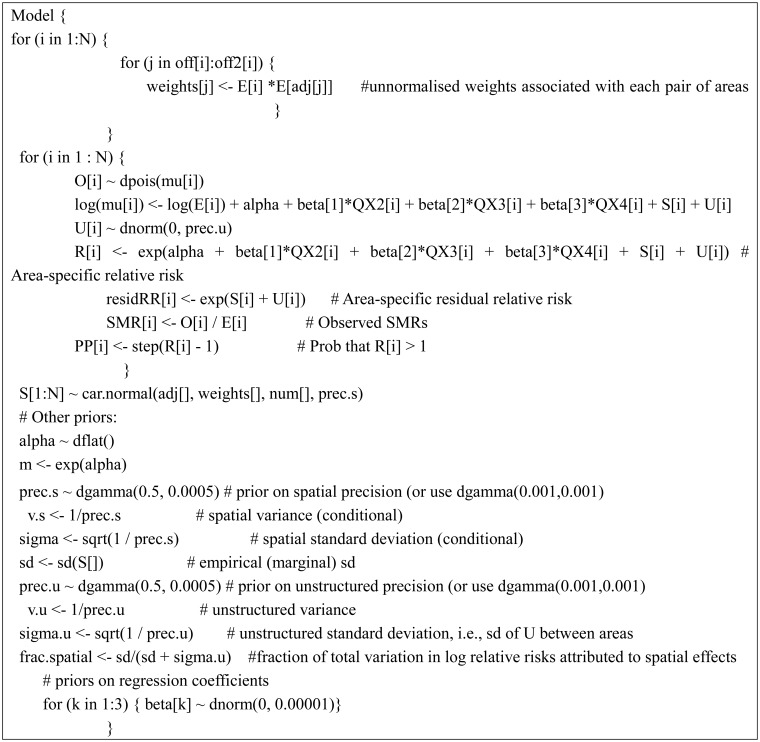
WinBUGS code for the best-fitted (final) model.

The final model (Equations (1), (5), (3), and (4)) was run using two chains. Convergence was monitored by visual examination of the trace plots of the samples for each chain, autocorrelation graphs and the Gelman-Rubin convergence statistics. Convergence occurred by 20,000 iterations. Each chain was then run for a further 10,000 samples giving 20,000 samples with acceptable Monte Carlo errors (< 5% of the sample posterior standard deviation). Based on Equation (5), we compared the results of model fit under the three approaches of specifying variable weights: (i) by senior population count; (ii) by senior population density; and (iii) by expected count of seniors' falls. The DICs for (i), (ii), and (iii) are 1,553, 1,558, and 1,547, respectively. Equation (5) with variable weights by expected count gives the smallest DIC suggesting that variable weights by expected count fits the data better. Sensitivity analysis using the different hyperpriors gives the same results. Figure 3 shows the WinBUGS code for this best-fitted model.

Ratios of posterior means of estimated risks for individual areas from GeoDa's incorrect and our correct adjacencies information have a mean of 1.00, standard deviation of 0.04, and maximum of 1.44. The estimated risks for Area #5 in Figure 2 based on GeoDa's incorrect and our correct adjacencies information are 1.21 (95% C.I.: 0.89, 1.59) and 1.28 (95% C.I. 0.94, 1.67), respectively. DICs from variable weights of expected falls (E_i_ x E_j_) using the incorrect (from GeoDa or the USGS program) versus correct adjacencies information differ by no more than three, suggesting that incorrect adjacencies information in this study has not caused a serious problem in risk estimations and model fit.

Table 1 compares the regression results of variable weights of expected count versus equal weights based on correct adjacencies. Importantly, occupied private dwelling requiring repairs, specifically, X_1:75_, is significant (nonzero regression coefficient at the 95% credible interval) under equal weights, but insignificant under variable weights by expected count. The fraction of spatial random effects, which represents the relative contribution of spatial versus unstructured heterogeneity to total between-area variation in log relative risks, is slightly higher for variable weights. This indicates that the fraction of total variation in log relative risks attributed to spatial effects after controlling for the covariates of dwelling and income is slightly higher for variable than equal weights. In a convolution model, theoretically, the two random effects (S_i_ and U_i_) contribute to twice the number of small areas of parameters; however, with spatial dependence and the inclusion of prior distribution specified by the ICAR model (different for equal and variable weights), both of which induce dependence between parameters, the number of parameters is reduced. Table 1 shows that the number of (effective) parameters from variable weights is six (=199–193) more than that of equal weights.

**Figure 4.**
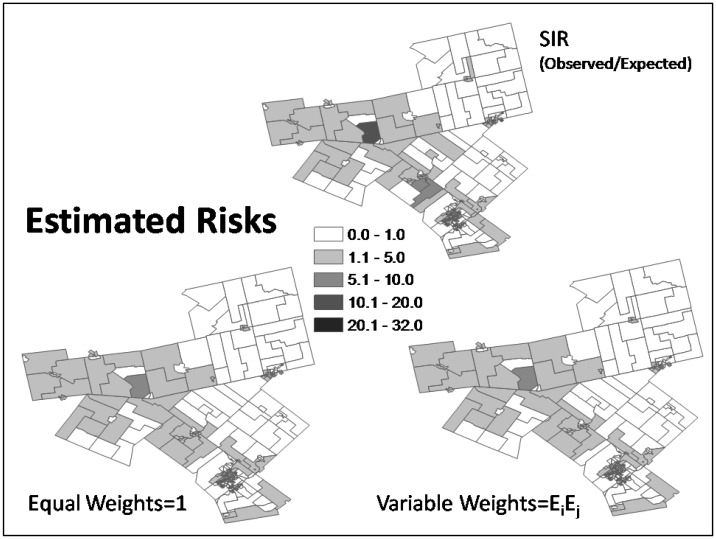
Comparing three risk maps: 1) standardized incidence ratios (SIR), 2) estimated risks from equal weights, and 3) estimated risks from variable weights of expected count.

To compare the results of estimated relative risks from equal weights and weights by expected count, we plotted three risk maps in Figure 4:1) crude standardized incidence ratios (SIRs, given by the observed count divided by the expected count of seniors' falls, i.e., estimated risks without smoothing); 2) estimated risks from equal weights; and 3) estimated risks from variable weights by expected count. The latter two maps look similar and both are smoother than the SIR map. Table 2 suggests that overall, variable weighting has less smoothing effect compared to equal weighting. The mean of estimated risks from SIR, equal weights and variable weights are 1.29 (from 0 to 31.91), 1.11 (from 0.13 to 7.45), and 1.27 (from 0.19 to 24.80), respectively.

**Table 1.** Regression results of the final model under equal weights and variable weights of expected count in specifying ICAR prior.

	Equal Weights = 1	Variable Weights^a^=E_i_E_j_
Regression coefficient^b^, β_3_: % occupied private dwelling requiring repair, 75^th^ percentile	0.321 (0.056, 0.602)^c^	0.248 (-0.023, 0.534)
Regression coefficient, β_4_: median income of population 15 years and over before tax	0.020 (0.002, 0.037)	0.018 (0.002, 0.034)
Fraction of spatial random effects, S	0.546 (0.412, 0.685)	0.688 (0.518, 0.880)
Variance of S	0.309 (0.159, 0.477)	2.296 (0.310, 11.460)
Number of effective parameters, pD	193	199
DIC	1,558	1,547

Note:

a. Variable weights based on expected counts were calculated using indirect standardization for sex and age (65–69, 70–74, 75–79, 80–84, and 85+).

b. Regression coefficients of β_1_ and β_2_ (Equation (5)) are different but insignificant at the 95% credible intervals for all the models fitted, and thus are not reported above.

c. All 95% credible intervals are enclosed in parentheses.

**Table 2.** Descriptive statistics of the estimated risks from equal weights and variable weights of expected counts.

Relative risks	SIR = observed count/expected count	Equal Weights = 1	Variable Weights =E_i_E_j_
Mean (standard deviation)	1.29 (0.13)	1.11 (0.85)	1.28 (2.09)
Median	0.88	0.92	0.90
Min, max	0, 31.91	0.13, 7.45	0.19, 24.80

**Figure 5.**
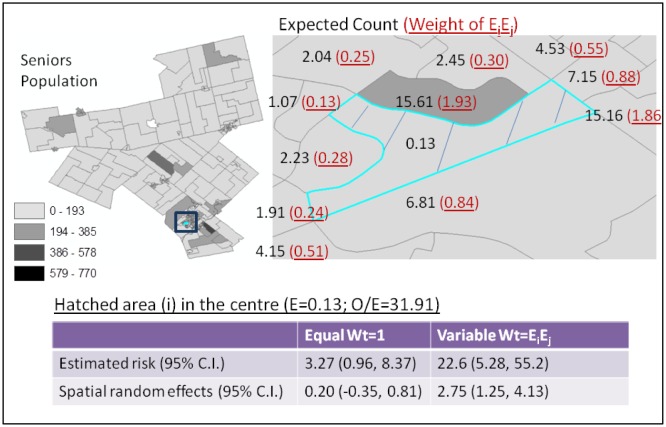
Comparing results of equal weights and variable weights of E_i_E_j_ for the area with the highest standardised incidence ratio. Note for the map on the right: 1. Hatched area in the centre has the highest SIR. It has 11 neighbors with different expected counts as shown. 2. The area in dark grey has higher (seniors) population at risk compared to other areas in this figure. 3. Weights of expected counts for determining the amount of information to borrow for estimating the risk of the centre area output from WinBUGS are underlined above.

**Figure 6.**
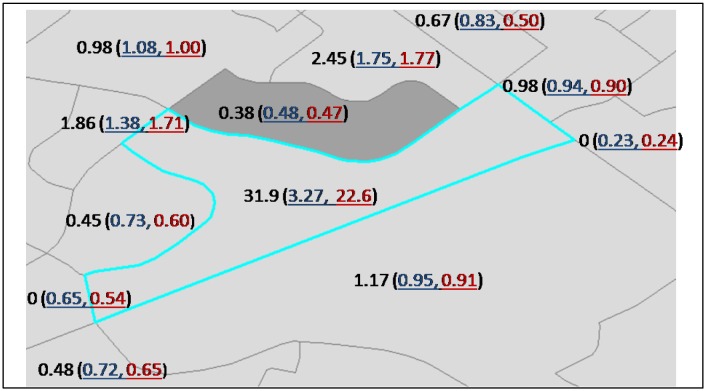
Smoothed risks by equal weights and variable weights of expected count compared to unsmoothed risks (SIR) for the area with the highest SIR and its adjacent areas. Note: 1. The three numbers shown above for each area are its SIR followed by its smoothed risks (first from equal weights of one then from variable weights of E_i_E_j_) underlined and in parenthesis. 2. Hatched area in the centre has the highest SIR (= 31.92). 3. The area in grey has higher (seniors) population at risk compared to all other areas above.

For individual areas, smoothing effect and results of its estimated risk are dependent on its neighboring information where strengths are borrowed for the estimation. For areas with low counts of observed case and expected count (due to low population-at-risk), their estimated risks from variable weights (E_i_E_j_) are not always closer to or farther away from the corresponding SIR compared to the estimated risks from equal weights nor with greater uncertainties. For example, the area with the highest SIR (hatched at the centre of the enlarged map on the right of Figure 5) has four observed incidents of falls with an expected count of 0.13. Its SIR is the highest (= 31.91) in the study region. Its estimated risks from equal weights and variable weights are 3.27 (95% C.I.: 0.96, 8.37) and 22.6 (95% C.I.: 5.28, 55.2), respectively. The estimated risks, both with huge uncertainties, are remarkably different with the risk estimated from variable weights closer to the SIR. Figure 6 compares the SIRs and smoothed risks from equal and variable weights for this area and its neighbors. The figure also indicates that estimated risks from variable weights are not always closer to the corresponding SIRs. Another area that also has low observed count (= 1) and expected count (= 0.75) with a SIR of 1.33 has estimated risks of 0.92 (95% C.I.: 0.23, 2.33) and 0.43 (95% C.I.: -0.01, 1.01) from equal weights and variable weights, respectively. So, for this area, the estimated risk from equal weights is closer to the SIR with greater uncertainty.

**Figure 7.**
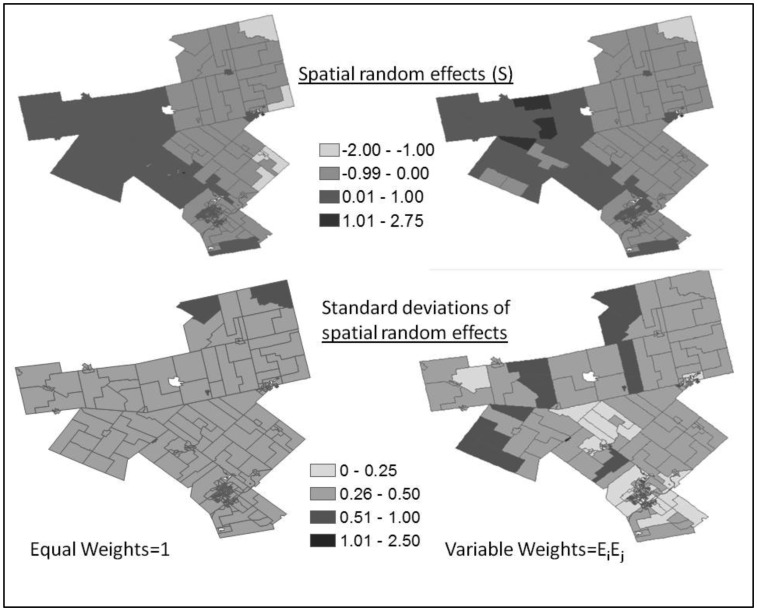
Spatial random effects and their standard deviations under equal weights (left maps) and variable weights of expected count (right maps).

In general, compared to equal weighting, variable weighting gives larger spatial random effects with larger standard deviations (see Figure 7). Spatial random effects from equal and variable weights have a minimum of -1.24 and -16.50, and a maximum of 1.17 and 2.75, respectively.

**Figure 8.**
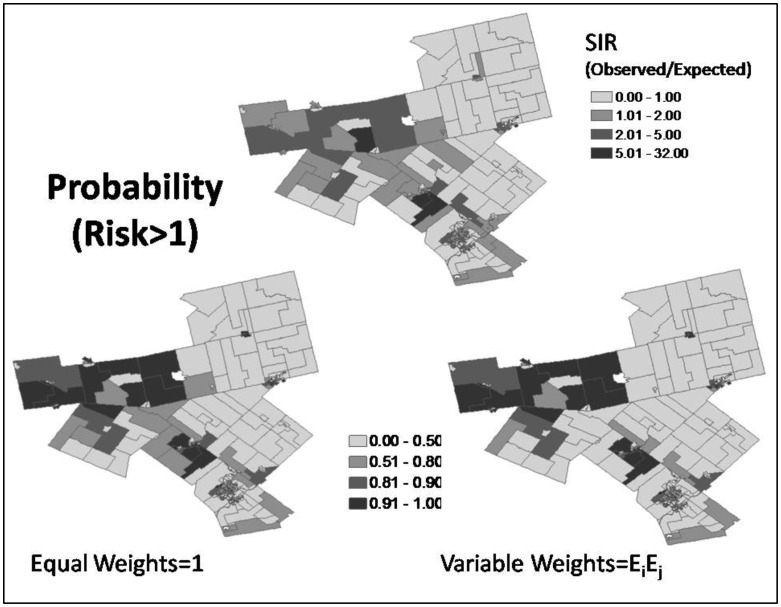
Crude risks of standardised incidence ratio (SIR) and probability of estimated risk greater than one from equal weights and variable weights.

Figure 8 shows the probability maps from equal weights and variable weights of expected count. Table 3 reports the number of areas with probability of risk greater than one tested at probability thresholds of 0.5, 0.75, 0.90, and 0.95 under expected-count weights versus equal weights.

**Table 3.** Number of areas with probability of estimated risk greater than one from equal weights versus variable weights.

	Number of areas with probability (Prob) of estimated risk greater than one (out of the 308 census dissemination areas)			
	Prob > 0.50	Prob >0.75	Prob >0.90	Prob > 0.95
Equal Weights: 1	110	71	49	38
Variable Weights: E_i_E_j_	105	73	51	41

## 4. Discussion

Specifications of spatial adjacencies and weights in ICAR modeling play an important role in small-are ecological studies [6],[19]. First, spatial adjacencies specified need to reflect true adjacencies between areas, but this has rarely been assessed. With incorrect information of spatial adjacencies, the spatial structure being modeled (both adjacency and weights) is wrong or different from what the model has assumed. Theoretically, two areas can be easily identified as adjacent if they have one or more boundary vertex with the same coordinates. However, in reality, different coordinates could be recorded for the same vertex for two adjacent areas in a digital map file resulting in common-vertex discrepancies. This could happen, for instance, if a common vertex of two adjacent areas was digitized as two different (although close) points from a paper map. As a result, areas that are supposed to be adjacent were not captured as such. Depending on the software used to generate adjacencies information from a digital map file, a threshold distance (tolerance) can be programmed within the package itself or set by the user to account for common-vertex discrepancies, but setting a threshold or developing an intelligent algorithm that can handle automatically all such discrepancies for any digital maps without identifying wrongly common vertices is not a straightforward task. This could explain why different software packages produce slightly different adjacencies information from the same digital map. Incorrect adjacencies information could be more problematic for study regions with a small number of areas (as the percentage of error is then large). In this study, incorrect adjacencies have not resulted in a serious change in model fit probably because the number of areas is relatively large, containing 1,734 neighbors, and thus error, in terms of the total number of neighbors, was only three percent ((=1734-1682)/1734).

In this case study, compared to equal weighting, expected-count weighting (E_i_E_j_) gives better model fit. Under equal weights, information is borrowed from all neighbors equally, disregarding their demographic or other characteristics. In contrast, under expected-count (of E_i_E_j_) weights, more information is borrowed from neighboring areas with similar or more counts of population-at-risk, and minimal information from neighboring areas with low population-at-risk, while controlling for the varying degrees of risks for different age groups. Specifying equal weights seems inappropriate for this study of seniors' falls, and possibly in similar studies that involve varying risks and population-at-risk among adjacent areas, when areas with relatively high and low population-at-risk are adjacent to each other. Some areas can have no or very low population-at-risk (seniors), as in this case study. Most seniors are unlikely to visit areas with no seniors or low population even though they are adjacent to their area of residence according to the (artificial) census map. Census areas or boundaries have little to do with common risk factors and could have variations in terms of population density, seniors' population, and distribution of different seniors' age groups. Our data do not inform the exact locations of falls, except that about 57% of seniors' falls were at home, but for seniors, it is reasonable to assume that falls are near their home or in their neighboring areas where the grocery stores, and their friends or activities are. Instead of borrowing information equally from all neighboring areas by equal weights, we therefore chose to borrow more information from neighbors that have similar seniors' fall risk and seniors' population by using the expected count of seniors' falls (accounting for populations in different seniors' age groups) as the variable for assigning (spatial) weight to two adjacent areas. Demographic variables, direct from Census, like population count or population density, for variable weights can control for varying population distribution, but would not be able to control for varying risks from different (seniors') age groups. However, they were also tested in this study for sensitivity analysis understanding that a different variable could produce different weights and thus results. Indeed, when we tried using the variables of seniors' population density and population count to derive the weights, model fits were found inferior to weights by expected count.

Over-estimation of significance of risk factors and over-smoothing of risk estimates are two critical concerns about equal (spatial) weights in this study. The regression coefficient of the percentage of occupied private dwelling that is significant under equal weights becomes insignificant at the 95% credible interval (Table 1). Both weight models have controlled for deprivation (using income as a covariate) with a positive association identified. These findings of associations about seniors' falls need further investigations, for instance, by using multilevel models to account for individual and environmental factors and non-linear models with interaction effects, which are beyond the scope of this case study that investigates spatial adjacencies and weights.

In this case study, smoothing of risks is generally less under expected-count weights than equal weights; however, expected-count weights has not resulted in estimated risks that are always closer to the SIR or with greater uncertainties for areas with low at-risk population or expected counts. Under variable weights of expected count, the strength borrowed is dependent on the expected counts of its neighbors, which in turn are dependent on the population of each of the senior age groups of the ten strata of the neighbors as well as each of the associated (injury) rates of the ten strata. For equal or similar rates in different stratum of senior age groups, the higher the corresponding senior population the neighboring area, the more strength to be borrowed from it. Expected-count weighting allows areas with extreme risk surrounded by areas that have non-extreme high risk to retain more of their characteristics of high risk. Over-smoothing by equal weights, as illustrated by the area with the highest SIR in Figures 5 and 6, is a concern. Avoiding the introduction of artefacts and autocorrelation in the resulting (smoothed) map is important [3]. Mapping probabilities of relative risk greater than one provides information about clustering of high-risk areas. The probability maps from equal weights and variable weights of expected count in Figure 8 look similar; however, Table 3 reveals that the number of areas with probability of risk greater than one is more from expected-count weights when tested at probability thresholds of 0.75, 0.90, and 0.95.

Results of spatial random effects provide useful insights. Spatial random effects (S) and their standard deviations shown in Figure 7 appear smoother and smaller under equal than expected-count weights. In Figure 7 (bottom right map), the larger standard deviations of S on the western side under expected-count weights are due to the spatial variation of risks, as seen in their SIRs, of their respective neighboring areas (see Figure 4, top map). The spatial random effects (S) model overdispersion or spatial autocorrelation in the data that persists after adjusting for available covariate information. Their estimations and uncertainties are affected by the way the spatial structure is specified. They have an important impact on the significance of the covariate(s) in the model and model fit (DIC). The more uncertain the spatial random effects, the less certain about the significance of the covariates, as indicated in this study - When we changed spatial weights from equal to expected-count, variance of spatial random effects became larger and the regression coefficient of private dwelling requiring repair became nonzero at the 95% credible interval (see Table 1), however DIC became smaller, indicating that the method of expected-count weights fit the data better.

Impact of the variable used to specify spatial weights on model fit and complexity (effective number of parameters or model parsimonious) should be explored. In this study, the model of variable weights of expected count that diminishes the significance of a covariate contributes to an extra six parameters as pD for the variable and equal weights are 199 and 193, respectively. However, its smaller DIC (1547 compared to 1558 from equal weights) suggests that the extra six parameters are worth it. To the extreme, when variable weights results in a low DIC but with effective parameters closely approximate the actual number of parameters, then the variable so identified that can capture most of the spatial variation of the dataset could be more meaningful than the (complex) model. Adjacent areas could have very different risk profiles that are measurable by the variable.

## 5. Conclusion

In Bayesian hierarchical spatial modeling, the ICAR model with prior information of adjacencies and spatial weights has been considered as one of the best choices available to smooth out random variation unrelated with underlying risk and stabilize estimated risks in small-area analysis [20]. This case study of seniors' falls using the ICAR model provides some evidence of incorrect adjacencies information caused by deficiencies in a digital map. Findings also suggest that equal weights could result in incorrect identification of risk factor(s) and over-smoothing of risks when geographical variation of population-at-risk (seniors in this study) exists. Weighting that uses the variable of expected count gives better model fit and better account for variation of the distribution of population-at-risk and risks of falls. In small-area analysis, sensitivity of results to the choice of spatial weights in ICAR modeling, especially in complex urban settings where the risk of injuries or adverse health outcomes is likely to exhibit more localized spatial structure, is worth considering.

Concerns over the methodologies of specifying spatial structure in small-area analysis is not new but an important issue that has yet to be addressed satisfactorily. The traditional use of equal weights in specifying spatial weights, which disregards the varying risk profiles and strengths of relationship between areal units, requires careful considerations. Variable weights using expected count outperforms equal weights in this case study, but this finding requires further investigations for applications in general. Further research that compares stochastic and non-stochastic approaches of variable weights through simulation studies, for instance, and explores approaches that allow the data to inform on the appropriate spatial weights to be specified is recommended.
